# Recreational Nitrous Oxide Causing Deep Vein Thrombosis and Subacute Combined Degeneration: Whip It Real Good

**DOI:** 10.7759/cureus.65155

**Published:** 2024-07-22

**Authors:** Megan E Lander, Skye J Lander, James Park, Chul Chae

**Affiliations:** 1 School of Medicine, California University of Science and Medicine, Colton, USA; 2 Radiology, Arrowhead Regional Medical Center, Colton, USA

**Keywords:** cobalamin deficiency, hypercoagulability, homocysteine, vitamin b12 deficiency, deep vein thrombosis, subacute combined degeneration, whippets, whippits, whip-its, nitrous oxide

## Abstract

Nitrous oxide (N_2_O) has been thought to be a harmless recreational substance by public perception, but it has been linked to subacute combined degeneration (SACD) due to induction of a functional vitamin B12 deficiency via oxidation and inactivation of the cobalt ion in its molecular structure. N_2_O has been rising in popularity due to several factors including accessibility, low cost, and low perceived risk, leading otherwise healthy people to develop what used to be a neurological disease experienced by select patient populations with dietary restrictions or medical conditions leading to low levels of vitamin B12. Vitamin B12 plays a crucial role in many cellular processes, and loss of functional vitamin B12 cannot be detected by measuring it directly. Substrates from its metabolic pathways such as homocysteine and methylmalonic acid must be measured to check its functional status. Vitamin B12 deficiency also leads to a hypercoagulable state due to the build-up of homocysteine in the blood. We present the case of a 26-year-old male who had reportedly used N_2_O for six months leading to SACD and a popliteal deep vein thrombosis. The options for treatment are abstaining from substance use and vitamin B12 supplementation; however, full recovery after SACD develops is unlikely and patients may be left with permanent neurological dysfunction from N_2_O use.

## Introduction

Nitrous oxide (N_2_O) saw its first use in 18th-century Britain for “laughing gas parties” after its first procurement in 1772. Its medical indications were quickly found as the odorless and colorless gas exhibited profound anesthetic and analgesic effects [1]. Currently, N_2_O is rarely used for analgesia and anesthesia in medical settings but is commonly commercially available as compressed gas used in food and entertainment products. It has been used recreationally, often inhaled from whipped cream canisters, hence the slang term spelled “whippets,” “whippits,” or “whip-its.” The 2016 National Survey on Drug Use and Health found that 9.1% of all participants tried inhalants at some point during their lifetime, wherein N_2_O constituted the highest proportion of inhalants at 48.6% of total inhalant use [2]. Varying sizes of canisters of pure N_2_O are widely available at a low cost, contributing to more widespread use in young adults in recent years.

N_2_O causes a functional vitamin B12 deficiency which can precipitate subacute combined degeneration (SACD), a disorder in which the dorsal and lateral columns of the spinal cord are affected. This induced B12 deficiency occurs through oxidation of the cobalt ion within cobalamin converting vitamin B12 from an active monovalent form to an inactive divalent form [1,3]. This process renders the vitamin B12 complex inactive, thus stores may remain normal with absent or minimal coenzyme activity. A secondary mechanism by which N_2_O can precipitate acute onset peripheral neurotoxicity is through direct neural damage from transient ischemia with inhalant use [1]. A study by Largeau et al. continued this idea and stated that there may be no “safe” recreational dose as low intermittent dose exposure may be sufficient to cause neurological damage. It was also found the median onset from the start of N_2_O use to neurological symptoms was six months [4]. Supplementation for prophylaxis of SACD while a patient is still actively using N_2_O therefore may not improve symptoms, and intramuscular vitamin B12 is considered the gold standard of treatment over oral repletion [1].

Vitamin B12 is a coenzyme for methionine synthase in the methionine cycle which forms homocysteine as an intermediate. A lack of vitamin B12 activity can lead to a buildup of homocysteine, and hyperhomocysteinemia has been shown to produce a hypercoagulable state, increasing the risk for venous thromboembolism and its sequelae [5]. In vitro, homocysteine has been associated with a reduction in binding sites for tissue plasminogen activator (tPA), causing a 60% decrease in tPA activity thus promoting a prothrombotic effect at the surface of the endothelial wall [6]. Homocysteine levels can be drastically elevated in individuals who use N_2_O due to a functional vitamin B12 deficiency and subsequent decreased methionine synthase activity.

This paper aims to contribute further to the current body of literature, dispel the perception that N_2_O is a harmless substance, and elucidate some of the more common and rare adverse effects this intoxicant can precipitate.

## Case presentation

We present the case of a 26-year-old male with a past medical history of obesity, type 2 diabetes mellitus, and asthma presenting to the Emergency Department (ED) for symmetrical weakness, numbness, and pain in his bilateral lower extremities for one month. He had been seen the same day at an outside hospital (OSH) ED for the same complaint. At the OSH, the patient was found to have a mildly decreased vitamin B12 level at 199pg/mL (OSH N = 200-1,240pg/mL), increased methylmalonic acid (MMA) of 19.06nmol/mL (OSH N ≤ 0.40nmol/mL), folate levels were reported as "within normal limits," as well as a partially occlusive left popliteal deep vein thrombosis (DVT) found by Doppler ultrasound and confirmed by left lower extremity CT angiogram. He was started on Eliquis 5mg BID, given a 1,000mcg vitamin B12 injection, and prescribed 1,000mcg oral vitamin B12 daily. He was discharged home but presented to our ED one hour later due to his inability to care for himself at home secondary to lower extremity weakness and paresthesias preventing ambulation.

**Figure 1:**
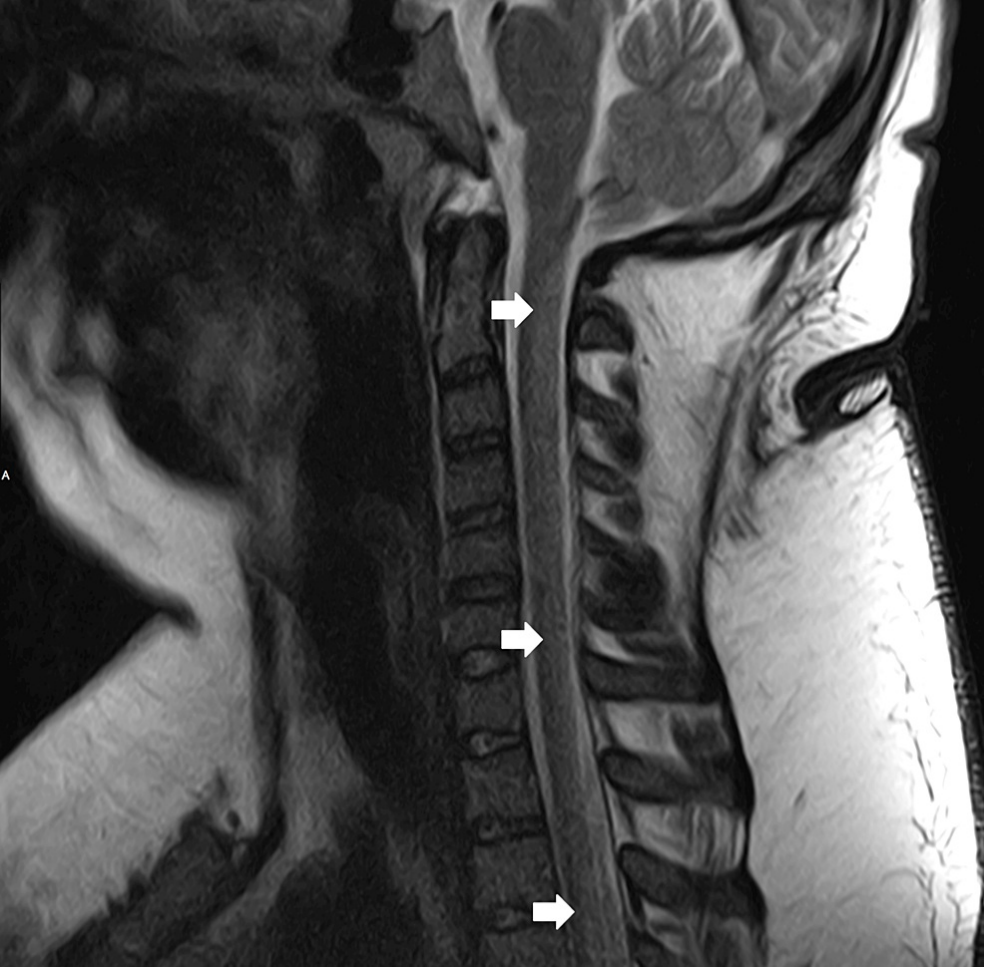
T2-weighted MRI of the cervical spine showed diffuse central and posterior cervical spinal cord signal abnormality, shown at arrow tips, consistent with subacute combined degeneration.

**Figure 2:**
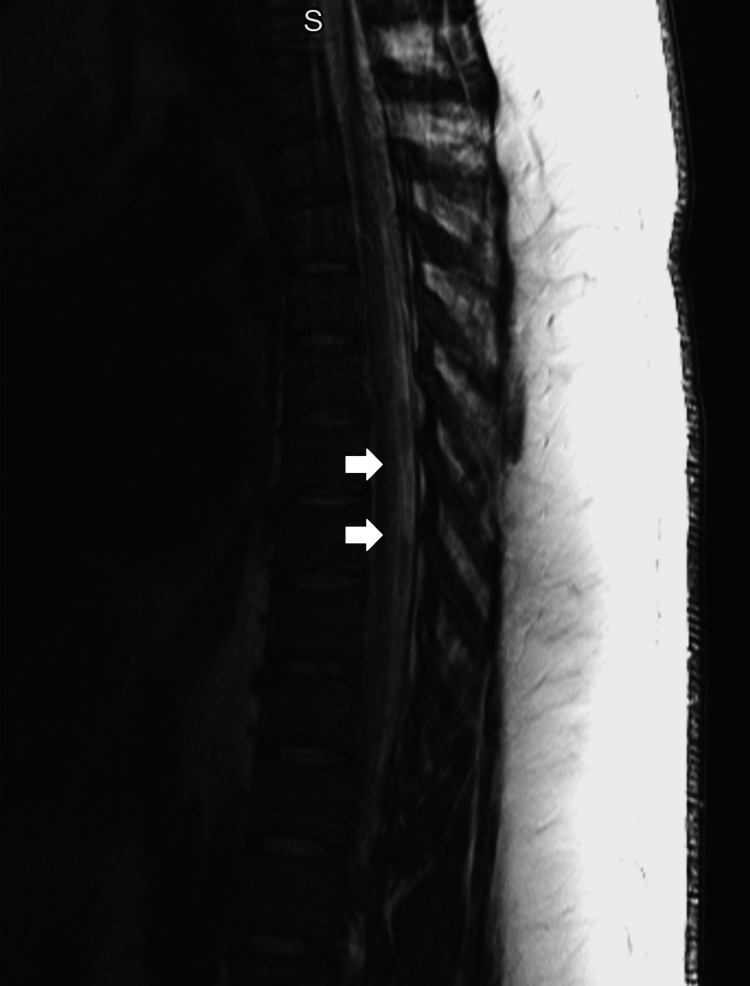
T2-weighted MRI of the thoracic spine (T-spine) showed diffuse central and posterior thoracic spinal cord signal abnormality, delineated by arrow tips, terminating near the lower end of the T-spine. A minor overlying artifact is present due to body habitus and motion near the superior T-spine.

The patient stated he had intermittently used N_2_O recreationally for the last six months with neurological symptom onset one month prior to admission. He reported sharing a 10lb N_2_O cylinder with a couple of friends every other weekend until the onset of noticeable neurological symptoms consisting of bilateral leg numbness, tingling, pain, and weakness up to his shins three weeks before presentation. He reported that his friends who also participated in using N_2_O had also recently begun to experience similar neurological symptoms. On physical exam his heel-to-shin testing showed ataxia bilaterally, Achilles and patellar reflexes were absent bilaterally, Romberg was not tested due to inability to stand, and the patient was found to have 3cm x 5cm bilateral, darkly pigmented medial leg lesions reportedly from skin contact with the freezing cold N_2_O cylinder during use. His vitamin B12 levels were now >2,000pg/mL (N = 250-1,100pg/mL), MMA was 2,759nmol/L (N = 87-318nmol/L), ESR was 37mm/hr (N = 0-10mm/hr), hemoglobin trended around 12.8g/dL (N = 13.0-17.0g/dL), hematocrit trended around 40% (N = 41-53%) with an MCV ranging from 90fL to 91fL (N = 80-100fL). CSF studies were unremarkable. MRI lumbar spine showed degenerative disc disease at L5-S1 without significant spinal cord compression. T2-weighted MRI showed central and posterior cervical and thoracic spinal cord signal enhancement (Figures 1-2) suggestive of transverse myelitis versus SACD, but given the patient’s clinical picture and history, SACD was higher on the differential. Vitamin B12 supplementation and anticoagulation were continued while the patient was admitted with improved neurological symptoms throughout his hospitalization. Reported paresthesias decreased in severity and regressed as they went from being in bilateral feet to bilateral toes. The patient’s lower extremity weakness continued to improve throughout his stay until the patient could stand on his own at discharge. The patient was given instructions for follow-up, counseled on substance cessation, and discharged after nine days of admission.

## Discussion

Because N_2_O induces a functional B12 deficiency, measured B12 levels do not necessarily correlate with SACD symptoms, severity, or resolution in these cases. Notably, this nonfunctional vitamin B12 may alter the expected lab values as many reports of N_2_O-induced SACD have presented with normal-to-low vitamin B12 levels and mild normocytic anemia [1,3,7]. In our case, the initial serological workup illustrated mildly low vitamin B12 levels initially which were corrected with vitamin B12 supplementation, as well as a mild normocytic anemia, a consistent finding in SACD from N_2_O. Pre-existing subclinical vitamin B12 deficiency is also more likely to evolve into SACD with lower dosages of N_2_O use, wherein even a single use can precipitate symptoms [8]. Several factors may lead to subclinical vitamin B12 deficiency and should increase physician’s index of suspicion for SACD secondary to N_2_O such as malabsorption syndromes, gastric acid suppressants, metformin use, and vegan diets, in addition to other patient factors such as male sex and being between the ages of 16 and 30. For patients with these risk factors, the dosage and frequency of N_2_O use become very important and the clinical index of suspicion for SACD should be fairly high with new-onset neurological deficits [7]. Of note, our patient’s high BMI may be another risk factor for SACD, due to obesity’s strong inverse relationship with vitamin B12 in young adults. This is thought to be due to epigenetic modification from increased adiposity and may have precipitated our patient’s presenting symptoms [9]. In SACD secondary to N_2_O, laboratory analysis typically shows high MMA and homocysteine levels, while T2-weighted MRI may show hyperintensity of the dorsal columns most commonly of C1-C5 [1,3,7].

A lack of functional vitamin B12 prevents methionine synthase from converting homocysteine to methionine, leading to excessive homocysteine. Consequently, studies have seen the effects of hypercoagulability secondary to hyperhomocysteinemia in this patient population. Notably, a young male patient with hyperhomocysteinemia secondary to N_2_O use suffered from a central pulmonary embolism, illustrating the effects hyperhomocysteinemia has on endothelial dysregulation and subsequent hypercoagulability [5-7]. Similarly, our young patient developed a left popliteal DVT, his only other risk factor being obesity [10]. Homocysteine levels were not checked directly in our patient’s case, but MMA levels were extraordinarily high, indicating a backup of substrates in the methionine cycle, inferring a build-up of homocysteine as well.

Of note for physicians, the terms canister and cylinder may be used synonymously, but one typical cylinder is equivalent to 75 canisters of N_2_O. Both the route and frequency should be evaluated for all patients with N_2_O intake [8]. Remarkably, excess N_2_O poses the acute danger of a decreased response to hypercapnia and hypoxia which may lead to a lack of awareness of respiratory status and subsequent asphyxiation [11].

The current treatment of SACD secondary to N_2_O use involves the immediate cessation of the offending agent, an initial intramuscular administration of 1mg vitamin B12, and a subsequent two-week course of 1mg vitamin B12 injections on alternating days, as vitamin B12 administration is recommended with this schedule until there is no further neurological improvement [1,8]. This was the case for our patient, whose symptoms improved clinically throughout his hospitalization with substance abstinence and B12 supplementation. Partial to full resolution can be achieved within 14 days to 21 months depending on the severity of symptoms [12]. Unfortunately, most patients are likely to have continued neurological deficits, as one study showed only 14% of SACD patients achieved full neurological symptom resolution with vitamin B12 supplementation [13].

## Conclusions

This case encompasses what may be considered a classic picture of N_2_O-induced functional B12 deficiency, which led to this patient's subsequent SACD and unprovoked DVT. Pre-existing low levels of B12 from diet and lifestyle may predispose patients to the development of SACD with the use of N_2_O, with as little as a single use. N_2_O is erroneously believed to be a harmless substance and is gaining in popularity due to this public perception, its availability, and relatively low cost, leading to an increase in SACD in people with otherwise no risk factors for vitamin B12 deficiency. The recovery from SACD after N_2_O use may never be complete, and patients may be left with neurological deficits in the long term. Functional vitamin B12 deficiency can also lead to a hypercoagulable state via the accumulation of homocysteine and resulting thrombosis, which may lead to devastating effects if not promptly treated. Substance abstinence and vitamin B12 supplementation are the current mainstays of treatment for N_2_O-induced SACD.
